# Improvement of Blood Parameters of Male Rats Exposed to Different Injection Doses of Liquid Chlorophyll

**DOI:** 10.7759/cureus.36044

**Published:** 2023-03-12

**Authors:** Yersultan D Tagauov, Abdelghafar M Abu-Elsaoud, Zhanna T Abdrassulova, Sultan T Tuleukhanov, Nurdana N Salybekova, Gulnar Tulindinova, Faten Al-Abkal

**Affiliations:** 1 Department of Biophysics, Biomedicine and Neuroscience, Al-Farabi Kazakh National University, Almaty, KAZ; 2 Department of Biology, College of Science, Imam Muhammad Ibn Saud Islamic University, Riyadh, SAU; 3 Department of Botany and Microbiology, Suez Canal University, Ismailia, EGY; 4 Department of Biology, Khoja Akhmet Yassawi International Kazakh-Turkish University, Turkistan, KAZ; 5 Department of Biological Sciences, Pavlodar Pedagogical University, Almaty, KAZ; 6 Department of Specialized Pharmacies, Ministry of Health, Kuwait, KWT

**Keywords:** plant materials, chlorophyll, blood platelets, white blood cells, full blood count, blood component therapy

## Abstract

Introduction

Chlorophylls are natural pigments in our everyday diet, especially with customers' rising preference for more natural and healthful habits. The antioxidant capabilities of both classes of lipophilic substances have been researched since disrupting antioxidant equilibrium appears to be linked to the development of several diseases.

Methods

This research aimed to evaluate the effect of injection with chlorophyll (30 and 60 mg/ml) on enhancing the blood parameters of rats. Twenty-one white male rats were included in this study and divided into three groups: control, 30 mg/ml, and 60 mg/ml.

Results

Treatment with liquid chlorophyll significantly increased white blood cells (WBCs), red blood cells (RBCs), granulocytes, lymphocytes, hemoglobin (Hgb), hematocrit (Hct), mean corpuscular Hgb concentration (MCHC), and platelets. However, it nonsignificantly increased mean corpuscular volume (MCV). These results confirm a great increase in important hematological parameters in response to exogenous injectable chlorophyll with concentrations of 30 and 60 mg/ml and at two different time points, 14 and 28 days after injection. The platelet count was significantly (p<0.001) increased after 30 mg/ml and 60 mg/ml.

Conclusion

These results show a significant increase in important hematological parameters in response to exogenous injectable chlorophyll. The liquid chlorophyll is recommended to increase blood parameters and improve blood characteristics avoiding anemia.

## Introduction

Chlorophylls are natural pigments in our daily diet, especially with consumers' increasing tendency toward more natural and healthy behaviors [1]. Dietary chlorophyll can be found as chlorophyll a and chlorophyll b in fresh fruits and vegetables, and as metal-free pheophytins and pyropheophytins in thermally processed fruits and vegetables [2]. Chlorophyll in the form of underutilized greens in fresh vegetables, supplements, liquid solutions, extracts, or tablets can be used effectively as a healthy and beneficial nutrient supplement [3].

Chlorophyll is the most prevalent plant photopigment in nature, with chlorophyll-a (Chl-a) accounting for nearly 75% of the green pigments found in plants [4]. Chl-a is a totally unsaturated asymmetric macrocyclic molecule with a hydrophobic nature, which contributes to its poor solubility in hydrophilic fluids [4]. Therefore, Chl can be effectively used as a nutrient in the form of underutilized greens in fresh vegetables, supplements, liquid solutions, extracts, or pills; addition to micellar copolymers, such as P123, which has been shown to be biocompatible, is essential for in vivo and in vitro analyses, as they guarantee the monomerization of the hydrophobic PS and the maintenance of its photophysical properties [5].

Chl-a and its metabolites have been shown to build up in several tissues, including the liver and gut [6,7], which suggests that these organs might be affected by these compounds. Chlorophylls have several positive benefits, and one of these is antioxidant activity, which helps to prevent oxidative DNA damage and lipid peroxidation by decreasing reactive oxygen species (ROS) and chelating metal ions [4,8-10]. The chemical nature of porphyrin allows chlorophylls to function as hydrogen donors, stopping the chain process [11]. Chlorophyll and other pigments, mostly isolated from sea algae, were investigated for their biological functions and potential health advantages [9]. Natural pigments, especially chlorophylls, provide several health benefits. They have been shown to have anti-inflammatory, anti-obesity, anti-angiogenic, and neuroprotective properties [9].

This research aims to evaluate the effect of two concentrations of liquid chlorophyll in enhancing the hematological parameters of experimental rats, including blood features.

## Materials and methods

Liquid chlorophyll

The chlorophyll used is a liquid chlorophyll ES (Extra-strength), Nature's sunshine product, Inc., Spanish Fork, UT84660 (Nature sunshine, Inc., USA) [12]. Liquid chlorophyll ES is a water-soluble extract obtained from alfalfa by extraction of chlorophyll (sodium-copper salt of chlorophyll). Liquid chlorophyll is a concentrated source of both Chl-a and Chl-b in addition to several nutrients of natural origin, including beta-carotene, vitamins C, E, and K. It is also rich in minerals and trace elements. Twenty-one white male rats were included in this study and divided into three groups: control, 30 mg/ml, and 60 mg/ml (A0, A1, and A2). Blood parameters were measured after 14 and 28 days of injection.

Sample size calculations

**Figure 1:**
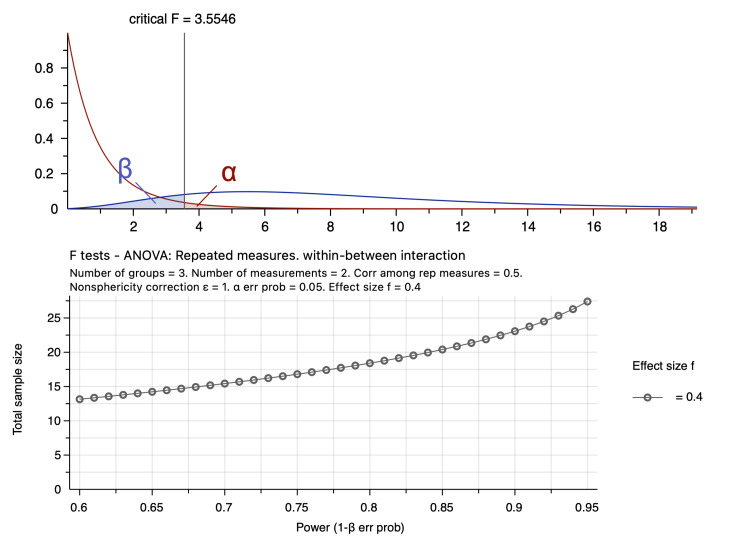
Sample size calculations using G*power software ANOVA, analysis of variance.

This research was performed to evaluate the effect of different concentrations of liquid chlorophyll on various blood parameters at two different time points, 14 and 28 days; a repeated-measures analysis of variance (ANOVA) has been proposed. A minimum total sample size of 21 white rats was sufficient to detect the effect size of 0.386, according to Cohen (1988), at a power (1-β=0.80) of 80% at a significance probability level of p <0.05, and a partial eta squared of 0.12. According to sample size calculations, each treatment group (A0, A1, and A2) including a control group, 30 mg/ml, and 60 mg/ml and time of investigations (T0, T1) would be represented by a minimum of seven rats, as shown in Table 1 and Figure 1. The sample size was calculated according to G*Power software version 3.1.9.6 [13-15].

**Table 1:** Variables of the study and interaction of variables (n=21)

Variables		Treatment group (A)			Total sample size
		A_0_	A_1_	A_2_	
Time of investigation (T)	T_0 _	A_0 _T_0_	A_1 _T_0_	A_2 _T_0_	21
	T_1_	A_0 _T_1_	A_1 _T_1_	A_2 _T_1_	
Total sample size		7	7	7	21

Animals and experiment design

According to sample size calculations, experiments were carried out on 21 non-purebred white male rats weighing 220-230 g. The experimental rats were divided into three groups: Group-I (A0) untreated control group received 10 ml of isotonic saline solution. Group-II (A1) experimental rats received 10 ml of 30 mg/ml liquid chlorophyll through tail-vein injection. Group-III (A2) Group-II (A1) experimental rats received 10 ml of 60 mg/ml liquid chlorophyll through tail-vein injection. Animals were fed and followed up regularly, including changes in body weight and other parameters following standard care. The peripheral blood sample was obtained 14 and 28 days following treatment. 

Hematological indices

Red blood cell (RBC) hematological indices, including RBC count, mean corpuscular volume (MCV), mean corpuscular hemoglobin (MCH), mean corpuscular hemoglobin (Hgb) concentration (MCHC), Hgb, and hematocrit (Hct), were determined using a Coulter Automated Cell Counter and various hematological indices were determined after 14 and 28 days of injection (Coulter AcT, Beckman Coulter, New York, NY, USA) [16].

Statistical analyses

Statistical analyses were applied to compare different treatment groups (A0, A1, and A2) at different times of investigations (T1 and T2). The data were collected, checked, revised, and organized in tables and figures using Microsoft Excel 2016. Data were subjected to outliers' detections and statistical normality tests to detect whether the data were parametric or nonparametric. Data were analyzed for descriptive statistics, both graphical and numerical descriptions. Inferential statistics for evaluating and comparing three different treatments (A0, A1, and A2) and time of investigations (T1 and T2) was performed by repeated-measures ANOVA or corresponding nonparametric analyses at significance levels of 0.05. ANOVA was followed by Duncan's multiple-range tests (DMRTs) to compare treatment groups or corresponding post hoc test for nonparametric data. Data analyses were carried out using the computer software Statistical Package for Social Sciences (SPSS ver. 28.0 for Mac OS; IBM Corp, Armonk, NY) [17].

## Results

Various measured blood parameters are presented in tables and figures as mean±standard deviation (±SD). The white blood cells (WBCs) count x 10^9^ in control, group-I (30 mg/ml), and group-II (60 mg/ml) showed an average (±SD) of 8.10±0.22, 8.52±0.16, and 8.77±0.08 after 14 days of injection, respectively; however, after 28 days, they showed an average of 8.10±0.22, 9.02±0.17, and 9.54±0.14; respectively.

**Figure 2:**
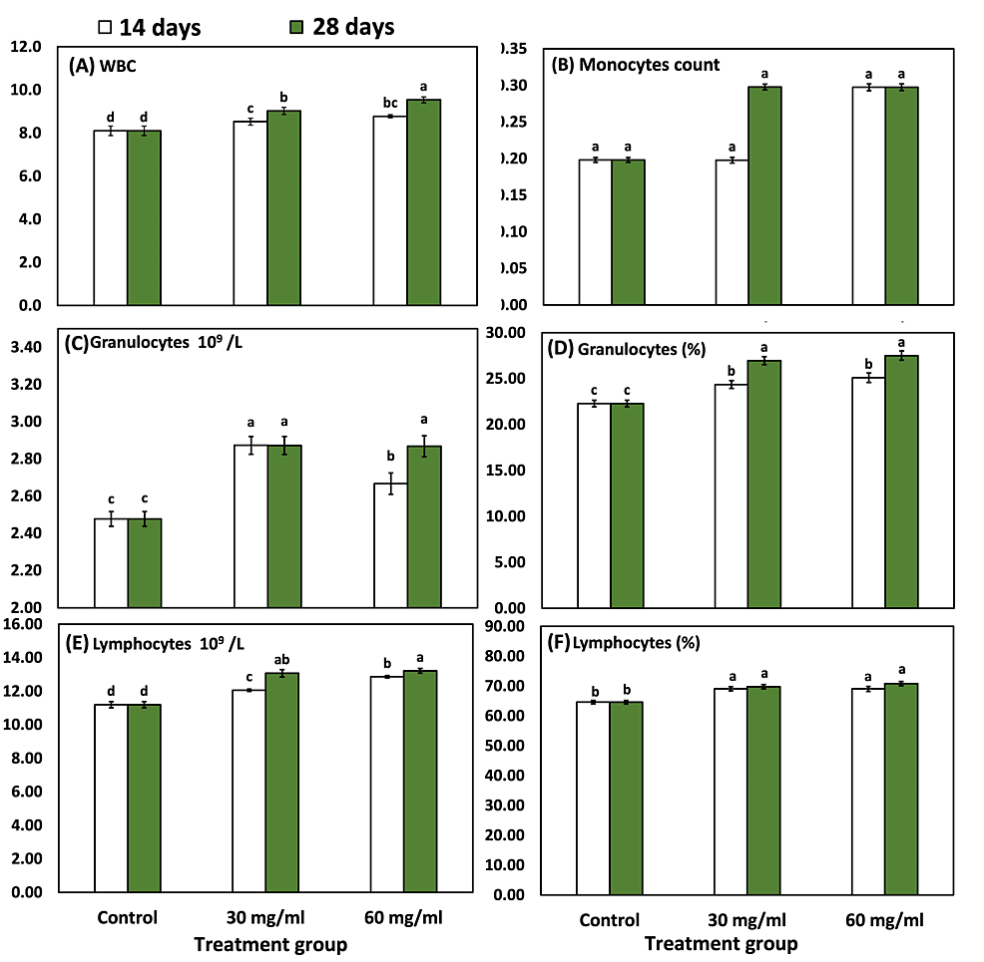
Various blood parameters in experimental male rats after treatment with 30 and 60 mg/ml of chlorophyll presented as mean and SE Bars followed by different letters are significantly different according to DMRTs. SE, standard error; DMRT, Duncan multiple-range test; WBC, white blood cells.

The WBC showed a highly significant increase after injection with liquid chlorophyll and also after the time of injection, as revealed by repeated-measures ANOVA (p<0.001) (Table 2; Figure 2). Moreover, the difference between groups at 14-day time points was highly significant (p<0.001) as revealed by one-way ANOVA. Also, the difference between groups at 28-day time points was highly significant (p<0.001) as revealed by one-way ANOVA.

**Table 2:** Various blood parameters in experimental male rats after treatment with 30 and 60 mg/ml of chlorophyll presented as mean and SD *, **, ***, significant at p<0.05, <0.01, <0.001; ns, nonsignificant at p>0.05. Means followed by different letters are significantly different according to DMRTs. Chl, chlorophyll; SD, standard deviation; DMRT,  Duncan multiple-range test; ANOVA, analysis of variance; WBC, white blood cells; RBC, red blood cells.

Parameter	Time	Mean±SD			Two-way ANOVA		
		Control	30 mg/ml	60 mg/ml	Chl	Time	Chl * Time
WBC	14	8.10±0.22 d	8.52±0.16 c	8.77±0.08 bc	0.001***	0.001***	0.162ns
	28	8.10±0.22 d	9.02±0.17 b	9.54±0.14 a			
Lymphocytes count (х10^9^/l)	14	11.19±0.19 d	12.06±0.07 c	12.86±0.07 b	<0.001***	<0.001***	0.003**
	28	11.19±0.19 d	13.07±0.22 ab	13.22±0.14 a			
Monocytes count	14	0.20±0.00 a	0.20±0.00 a	0.30±0.00 a	>0.05 ns	>0.05 ns	>0.05 ns
	28	0.20±0.00 a	0.30±0.00 a	0.30±0.00 a			
Granulocytes count (x10^9^/l)	14	2.48±0.04 c	2.87±0.05 a	2.67±0.06 b	0.013*	0.013*	0.013*
	28	2.48±0.04 c	2.87±0.05 a	2.87±0.06 a			
Lymphocytes (%)	14	64.60±1.04 b	69.09±1.23 a	69.04±1.49 a	0.520 ns	0.124 ns	0.466 ns
	28	64.60±1.04 b	69.78±1.26 a	70.80±1.21 a			
Monocytes (%)	14	1.59±0.03 e	2.08±0.03 c	1.98±0.04 d	0.007**	<0.001>	<0.001>
	28	1.59±0.03 e	2.78±0.03 b	3.08±0.04 a			
Granulocytes (%)	14	22.29±0.36 c	24.35±0.43 b	25.10±0.52 b	0.033*	<0.001>	0.758 ns
	28	22.29±0.36 c	26.95±0.43 a	27.51±0.51 a			
RBC х 10^12^/l	14	6.99±0.11 b	7.47±0.13 a	7.46±0.16 a	0.713	0.467 ns	0.713 ns
	28	6.99±0.11 b	7.52±0.14 a	7.60±0.16 a			

The lymphocytes (%) showed an average (±SD) of 64.60±1.04, 69.09±1.23, and 69.04±1.49 after 14 days, and 64.60±1.04, 69.78±1.26, and 70.80±1.21 after 28 days of injection in control, group-I, and group-II (Table 2, Figure 2). Furthermore, monocyte count recorded an average (±SD) of 1.59±0.03, 2.08±0.03, and 1.98±0.04 after 14 days of injection, and 1.59±0.03, 2.78±0.03, and 3.08±0.04 after 28 days of injection. The change in monocyte count with chlorophyll injection was nonsignificant.

Granulocytes count also showed a significant increase with a 30 mg/ml dose of chlorophyll, as revealed by Duncan's multiple range test and one-way ANOVA. The granulocytes level recorded an average of 2.48±0.04, 2.87±0.05, and 2.67±0.06 after 14 days of chlorophyll injection; however, it recorded an average (±SD) of 2.48±0.04, 2.87±0.05, and 2.87±0.06 after 28 days of chlorophyll injection. The change in granulocytes with chlorophyll was highly significant (Table 2, Figure 2).

**Figure 3:**
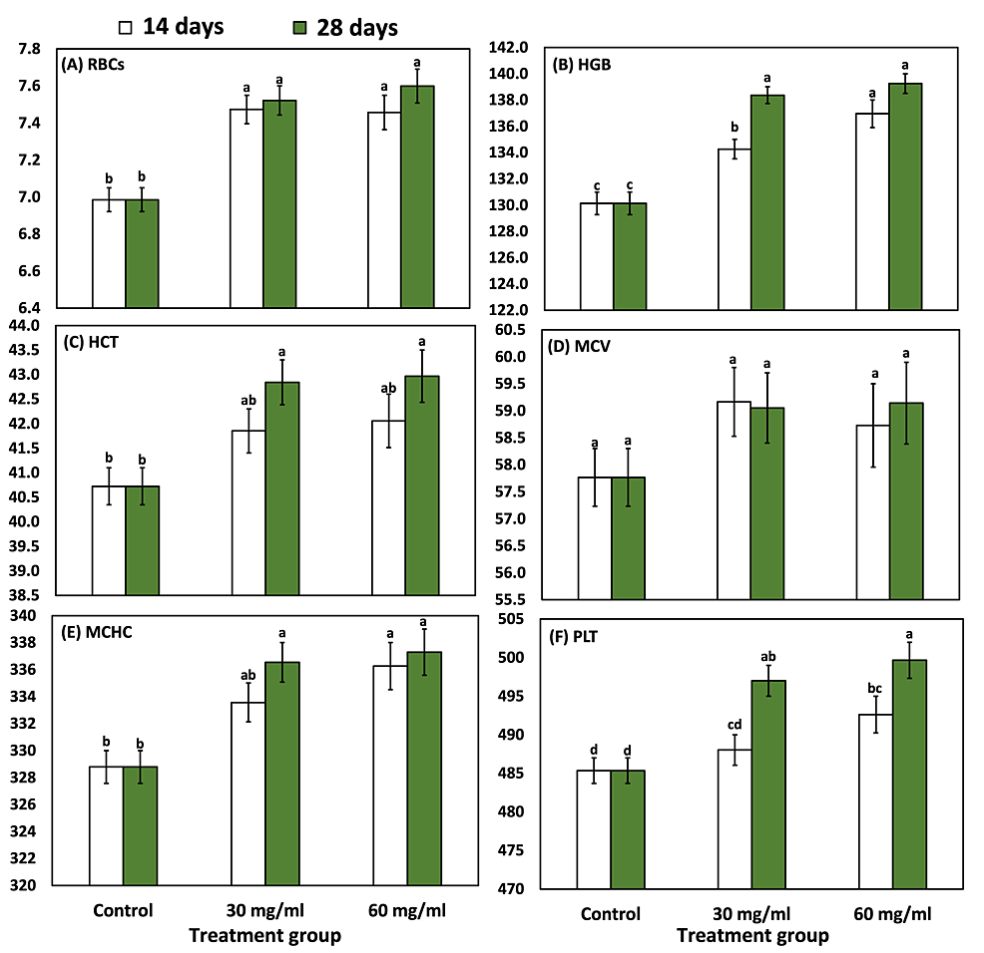
Various blood parameters in experimental male rats after treatment with 30 and 60 mg/ml. Bars followed by different letters are significantly different according to DMRTs. RBC, red blood cell; HGB, hemoglobin; HCT, hematocrit; MCV, mean corpuscular volume; MCHC, mean corpuscular hemoglobin concentration; PLT, platelet; DMRT, Duncan multiple-range test.

RBCs count (x10^12^/l) showed an average level in control, 30 mg/ml, and 60 mg/ml chlorophyll of 6.99±0.11, 7.47±0.13, and 7.46±0.16 x 10^12^/l after 14 days of chlorophyll injection, respectively. However, after 28 days it showed an average (±SD) of 6.99±0.11, 7.52±0.14, and 7.60±0.16 x 10^12^/l, respectively, for control, 30 mg/ml, and 60 mg/ml (Figure 3). The difference in RBCs was nonsignificant, as revealed by a two-way ANOVA. The HGB (g/l) level showed a significant increase with an increase in chlorophyll concentrations, as revealed by two-way ANOVA.

The HCT (%), MCV, MCH, MCHC, RDW, stable neutrophils, PCT (%), and basophils showed a nonsignificant response to exogenous injectable liquid chlorophyll, as revealed by a two-way ANOVA (Table 3).

**Table 3:** Various blood parameters are presented as mean and SD *, **, ***, significant at p<0.05, <0.01, <0.001; ns, nonsignificant at p>0.05. Means followed by different letters are significantly different according to DMRTs. Chl, chlorophyll; SD, standard deviation; HGB, hemoglobin; HCT, hematocrit; MCV, mean corpuscular volume; MCH, mean corpuscular hemoglobin; MCHC, mean corpuscular hemoglobin concentration; RDW, red cell distribution width; PLT, platelet; MPV, mean platelet volume; PCT, plateletcrit; DMRT, Duncan multiple-range test.

Parameter	Time	Mean±SD				Two-way ANOVA		
		Control	30 mg/ml	60 mg/ml		Chl	Time	Chl * Time
HGB (g/l)	14	130.13±1.50 c	134.26±1.29 b	136.95±1.81 a		0.050*	0.003*	0.294 ns
	28	130.13±1.50 c	138.37±1.10 a	139.25±1.29 a				
HCT %	14	40.72±0.65 b	41.85±0.78 ab	42.06±0.94 ab		0.731 ns	0.076 ns	0.945 ns
	28	40.72±0.65 b	42.84±0.79 a	42.97±0.92 a				
MCV (fl)	14	57.77±0.93 a	59.16±1.10 a	58.73±1.34 a		0.791 ns	0.828 ns	0.718 ns
	28	57.77±0.93 a	59.05±1.12 a	59.14±1.31 a				
MCH (pg)	14	19.62±0.31 b	19.88±0.37 ab	20.24±0.45 ab		0.303 ns	0.127	0.725
	28	19.62±0.31 b	20.38±0.38 ab	20.54±0.45 a				
MCHC (g/l)	14	328.79±2.10 b	333.56±2.49 ab	336.25±3.02 a		0.280 ns	0.217 ns	0.539 ns
	28	328.79±2.10 b	336.53±2.54 a	337.29±2.97 a				
RDW (%)	14	15.36±0.25 a	15.23±0.29 a	15.39±0.36 a		0.520 ns	0.074 ns	0.781 ns
	28	15.36±0.25 a	14.93±0.30 a	15.00±0.35 a				
PLT (х10^9^/l)	14	485.35±2.86 d	488.03±3.41 cd	492.61±4.13 bc		0.114 ns	0.003**	0.659 ns
	28	485.35±2.86 d	497.00±3.47 ab	499.66±4.06 a				
MPV (fl)	14	6.74±0.11 a	6.43±0.13 bc	6.71±0.16 ab		0.024*	0.096 ns	0.554 ns
	28	6.74±0.11 a	6.32±0.13 c	6.51±0.15 abc				
RDW	14	8.32±0.13 a	8.01±0.16 b	8.09±0.19 ab		>0.999 ns	0.060 ns	0.314
	28	8.32±0.13 a	8.31±0.16 ab	8.19±0.19 ab				
PCT (%)	14	0.33±0.01 a	0.35±0.01 a	0.34±0.01 a		0.226	0.226 ns	0.226 ns
	28	0.33±0.01 a	0.36±0.01 a	0.35±0.01 a				

The segmented neutrophils showed a highly significant difference with chlorophyll injection with concentrations of 30 and 60 mg/ml, as revealed by two-way ANOVA.

The lymphocytosis and neutrophilia were recorded in control, group-I, and group-II and were nonsignificantly changed with time or chlorophyll concentration. However, lymphopenia, neutropenia, eosinophilia, basophilia, and monocytosis were not recorded in any group or time points (Tables 4, 5).

**Table 4:** Various blood parameters presented as mean and SD. *, **, ***, significant at p<0.05, <0.01, <0.001; ns, nonsignificant at p>0.05. Means followed by different letters are significantly different according to DMRTs. Chl, chlorophyll; SD, standard deviation; ANOVA, analysis of variance; DMRT, Duncan multiple-range test.

Parameter	Time	Mean±SD				Two-way ANOVA		
		Control	30 mg/ml	60 mg/ml		Chl	Time	Chl * Time
Stable neutrophils	14	0.99±0.02 a	-0.01±0.02 a	-0.01±0.02 a		>0.05 ns	>0.05 ns	>0.05 ns
	28	0.99±0.02 a	-0.01±0.02 a	0.99±0.02 a				
Segmented neutrophils	14	24.92±0.15 b	25.90±0.17 a	21.88±0.21 c		<0.001***	<0.001***	<0.001***
	28	24.92±0.15 b	25.90±0.18 a	24.88±0.21 b				
Eosinophils count	14	0.00±0.00 a	1.00±0.00 a	1.00±0.00 a		>0.05 ns	>0.05 ns	>0.05 ns
	28	0.00±0.00 a	0.00±0.00 a	0.00±0.00 a				
Basophils count	14	0.00±0.00 a	0.00±0.00 a	0.00±0.00 a		>0.05 ns	>0.05 ns	>0.05 ns
	28	0.00±0.00 a	1.00±0.00 a	1.00±0.00 a				
Lymphocytes count	14	65.40±1.05 d	67.28±1.25 cd	69.13±1.51 bc		0.105	0.001***	0.529
	28	65.40±1.05 d	71.27±1.27 ab	72.14±1.48 a				
Monocytes count	14	0.99±0.02 a	1.99±0.02 a	0.99±0.02 a		>0.05 ns	>0.05 ns	>0.05 ns
	28	0.99±0.02 a	2.99±0.02 a	2.99±0.02 a				

**Table 5:** Lymphocytosis, lymphopenia, neutrophilia, neutropenia, eosinophilia, basophilia, and monocytosis are presented as median and mean *, **, ***, significant at p<0.05, <0.01, <0.001; ns, nonsignificant at p>0.05. Differences assessed by chi-square test.

All	Time (d)	Control		Treatment with chlorophyll				Chi-square
				30 mg/ml		60 mg/ml		
		Median	Mean	Median	Mean	Median	Mean	
Lymphocytosis	14	1	1	1	1	1	1	>0.05 ns
	28	1	1	1	1	1	1	
Lymphopenia	14	0	0	0	0	0	0	>0.05 ns
	28	0	0	0	0	0	0	
Neutrophilia	14	1	1	1	1	1	1	>0.05 ns
	28	1	1	1	1	1	1	
Neutropenia	14	0	0	0	0	0	0	>0.05 ns
	28	0	0	0	0	0	0	
Eosinophilia	14	0	0	0	0	0	0	>0.05 ns
	28	0	0	0	0	0	0	
Basophilia	14	0	0	0	0	0	0	>0.05 ns
	28	0	0	0	0	0	0	
Monocytosis	14	0	0	0	0	0	0	>0.05 ns
	28	0	0	0	0	0	0	

**Figure 4:**
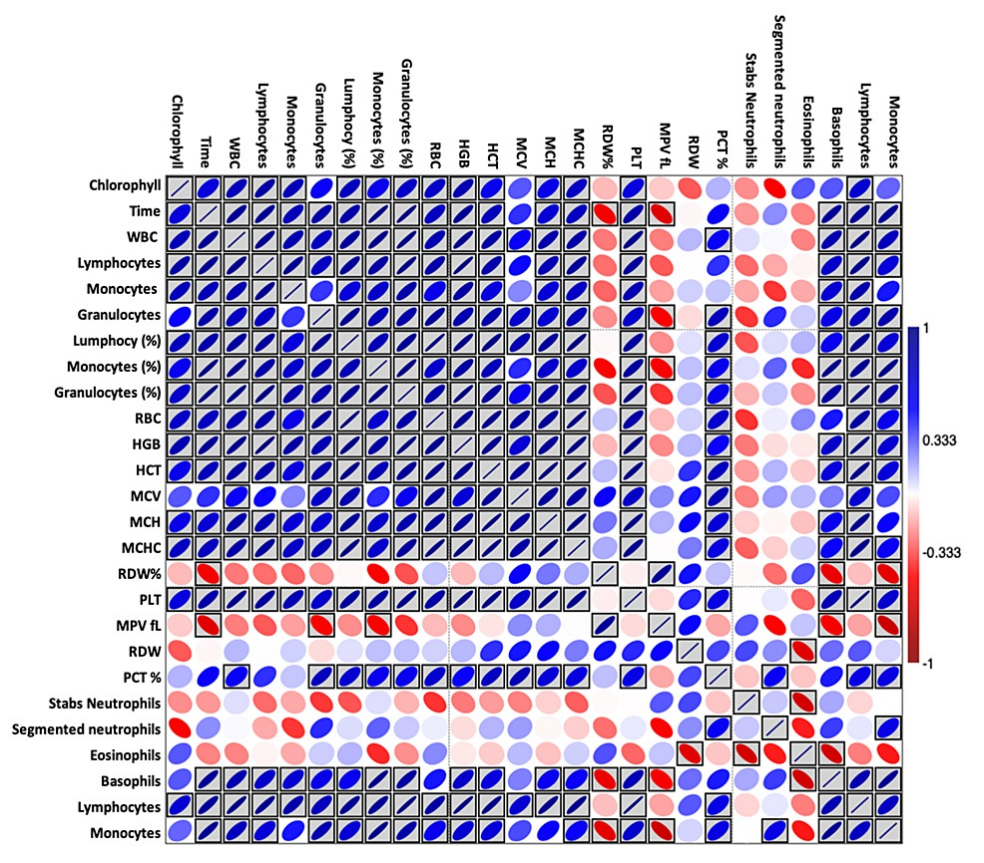
Heat map showing the interrelationships between variables. WBCs, white blood cells; RBC, red blood cell; Hgb, hemoglobin; Hct, hematocrit; MCV, mean corpuscular volume; MCH, mean corpuscular hemoglobin; MCHC, mean corpuscular hemoglobin concentration; RDW, red cell distribution width; PLT, platelet; MPV, mean platelet volume; PCT, plateletcrit; DMRT, Duncan multiple-range test.

Table 6 and Figure 4 present the relationship between exogenous injectable chlorophyll concentration and time versus various blood parameters presented as correlation coefficient (r) and two-tailed significance test (p-value). The chlorophyll treatment significantly and positively increased WBC (r=0.744; p=0.001***), lymphocytes х 10^9^/l (r=0.74; p=0.002**), monocytes (r=0.761; p≤0.001***), lymphocytes (%) (r=0.612; p=0.015*), monocytes (%) (r=0.546; p=0.035*), granulocytes (%) (r=0.635; p=0.011*), Hgb (g/l) (r=0.682; p=0.005**), MCH (pg) (r=0.52; p=0.047*), MCHC (g/l) (r=0.687; p=0.005**), PLT х 10^9^/l (r=0.649; p=0.009**), and lymphocytes (r=0.647; p=0.009**). Figure 4 represents a heatmap with the correlation coefficients, where blue color showed a positive correlation, red for a negative correlation, and boxed colors for significant correlations (Figure 4).

**Table 6:** The relationship between exogenous injectable chlorophyll concentration and time on various blood parameters is presented as a correlation coefficient (r) and two-tailed significance test (p-value) *, **, ***, significant at p<0.05, <0.01, <0.001; ns, nonsignificant at p>0.05. Means followed by different letters are significantly different according to DMRTs. WBCs, white blood cells; RBC, red blood cell; Hgb, hemoglobin; Hct, hematocrit; MCV, mean corpuscular volume; MCH, mean corpuscular hemoglobin; MCHC, mean corpuscular hemoglobin concentration; RDW, red cell distribution width; PLT, platelet; MPV, mean platelet volume; PCT, plateletcrit; DMRT, Duncan multiple-range test.

Variables	Chlorophyll concentration		Time	
	r	p-Value	r	p-Value
WBCs count	0.74	0.001***	0.91	<0.001***
Lymphocytes count (х10^9^/l)	0.74	0.002**	0.88	<0.001***
Monocytes count	0.76	<0.001***	0.76	<0.001***
Granulocytes x 10^9^/l	0.32	0.241 ns	0.75	0.001***
Lymphocytes (%)	0.61	0.015*	0.75	0.001***
Monocytes (%)	0.55	0.035*	0.94	<0.001***
Granulocytes (%)	0.64	0.011*	0.94	<0.001***
RBC х 10^12^/l	0.63	0.011*	0.68	0.005**
Hgb (g/l)	0.68	0.005**	0.84	<0.001***
Hct (%)	0.50	0.059 ns	0.68	0.005**
MCV	0.24	0.384 ns	0.38	0.161 ns
MCH (pg)	0.52	0.047*	0.68	0.005**
MCHC (g/l)	0.69	0.005**	0.71	0.003**
RDW (%)	-0.04	0.886 ns	-0.49	0.062 ns
PLT count (х10^9^/l)	0.65	0.009**	0.83	<0.001**
MPV	-0.03	0.917 ns	-0.59	0.020*
RDW	-0.33	0.224 ns	-0.01	0.983 ns
PCT (%)	0.10	0.719 ns	0.51	0.054 ns
Rod nuclear neutrophils	-0.15	0.588 ns	-0.15	0.588 ns
Segment nuclear neutrophils	-0.47	0.078 ns	0.27	0.331 ns
Eosinophils	0.30	0.270 ns	-0.30	0.270 ns
Basophils	0.30	0.270 ns	0.91	<0.001***
Lymphocytes	0.65	0.009**	0.86	<0.001***
Monocytes	0.25	0.369 ns	0.92	<0.001***

## Discussion

The liquid chlorophyll in this study showed a significant appositive correlation with WBCs, lymphocytes, monocytes, lymphocytes (%), granulocytes, RBCs, Hgb, Hct, MCV, MCH, MCHC, and blood platelets. These significant positive effects of chlorophyll concentrations tested indicate a high health benefit of using chlorophyll from biological sources. These results agree with those of Pangestuti and Kim [9] who listed various significant effects of natural pigments, including chlorophyll. However, it disagrees with the study by Cugliari et al. [18], in order to verify the effects of the protracted intake of chlorophyll on blood count parameters and iron levels in endurance athletes, investigating supposed anti-anemic properties. They reported no significant difference in blood parameters, including hemoglobin; however, they reported an increase in blood platelets [18].

The increase in platelet-related measures could positively influence endurance performance by reducing pain and fatigue. However, the supposed ergogenic effects and anti-anemic properties are recommended for further study [18]. Platelet-rich plasma has anti-inflammatory and anabolic effects [19] and several studies show its effectiveness in the healing process of muscle injury [20], tendon injury [21], and in the treatment of osteoarthritis [22]. A recent study shows a significant correlation between MPV and the running time in a half marathon [23], while in short-term performance at the maximum intensity, it appears to have no significant relationship between PLTS, MPV, and PDW with VO_2_Max, resistance, and running speed [24]. These results suggest that platelets may play a role in the medium- to a long-term performance by promoting the gradual release of growth factors and thereby relieving muscular pain and/or fatigue, or that MPV increase could be attributed to a more significant turn-over of platelets that could reflect other chronic physical adaptation without necessarily having a direct ergogenic effect. In this study, however, only the experimental group obtained a significant increase, indicating chlorophyll's role in modifying the above factor. 

The effect of chlorophyll on improving blood parameters includes antioxidant activities. Antioxidants may have a positive effect on human health as they can protect the human body against damage by ROS, which attack macromolecules such as membrane lipids, proteins, and DNA, leading to many health disorders such as cancer, diabetes mellitus, aging, and neurodegenerative diseases [25].

## Conclusions

The results of this study show a significant increase in some important hematological parameters in response to injectable chlorophyll including WBCs, lymphocytes count, monocytes count, lymphocytes (%), monocytes (%), and granulocytes (%), in addition to RBCs, Hgb, Hct, MCH, MCHC, and platelet counts. The liquid chlorophyll is recommended to increase blood parameters and improve blood characteristics, avoiding anemia. Further investigations are recommended to check the effects and side effects of using chlorophyll.
